# Physiological Influence of Alcohol*“The Physiological Influence of Alcohol.” By James Edmunds, M.D. Tweedie, Strand. 1874.

**Published:** 1874-02

**Authors:** 


*The Physiological Influence of Alcohol.**

This pamphlet is the substance of a paper read by Dr. Edmunds at a meeting of the British Medical Association in August last, and is full of interest, since the question of the use of alcohol is certainly one of the most important of the day. Dr. Edmunds, indeed, states that some one hundred and twenty millions are annually consumed in this country on alcoholic drinks.

Dr. Edmunds shows that pure alcohol is a powerful caustic, and that it does not answer the definition of food, not being innocuous to the system, or capable of entering into the construction of any living tissue. Cane sugar yields about fifty-one per cent. of alcohol, and the thermic power of a pound of sugar is equivalent to that of the alcohol contained in a pound of brandy; but sugar is completely oxydized in the system, whilst a very great portion of all the alcohol taken into the economy is excreted unchanged in the breath and urine.

Dr. Edmunds upon this point cites the opinion of Professor Frankland, that conclusive evidence is still wanting as to the oxydization of alcohol in the system; but there appears to be considerable probability that some at least is oxydized. Hence the food value of alcohol is truly very small.

In large doses, alcohol is a powerful narcotic, and more persons are killed by it than by all other poisons taken together. According to Dr. Edmunds, alcohol, chloroform, opium, and intense cold seem to lessen vital activity by retarding oxydation in the body,

* "The Physiological Influence of Alcohol." By James Edmunds, M.D. Tweedie, Strand. 1874.

just as the flame of a candle burns more dimly when the vapor of alcohol surrounds it.

It is said that alcohol in small doses increases the activity of the heart's action, and that it should therefore be given whenever the pulse is feeble. Dr. Edmunds submits that in a soundly organized animal the power exercised by the heart is an exact measure of the difficulty of keeping the circulation going. The real cause of the capillary current in animals is the same as that which moves the sap in plants, *i. e.*, the vital activity of the tissues themselves. Whilst, then, the sympathetic nerve is duly sensitive, reflex action urges on the heart to get rid of the blood, and thus keeps up a distended state of the arteriales, from which the tissues draw their blood supply. The real pace of the circulation is determined by the general molecular activity of the tissues, and not by the action of the heart.

In feeble and inactive states of the system, the heart should work very gently; and the administration of stimulants in a depressed or aged system, with a view to urge on the movements of the heart, is most irrational.

As a consequence of these views, Dr. Edmunds holds that the true and only object for which alcohol should be used as a medicine, should be in cases where convulsions are likely to supervene in children, or for retarding over-action in some stages of general disease, and he ends with drawing the following conclusions:

1. That all alcoholic beverages are ordinarily taken for the alcohol they contain, and would not be taken were the alcohol abstracted.

2. That the non-alcoholic constituents of these beverages may all be obtained in their most advantageous conditions, and at a fractional cost, in common-place articles of food.

3. That alcohol, if a food, is the most absorbable and rapidly diffusing food substance with which we are acquainted, and one, moreover, which is utilizable without effort from the digestive organs, but that in these respects an equal quantity of grape sugar would have nearly the same advantages.

4. That the food value of alcohol cannot exceed that of the sugar from which it is manufactured, and therefore, to use it as an ordinary article of food, must, at the present relative prices of sugar and alcohol, be a vast monetary extravagance.

5. That a considerable portion of the alcohol is excreted from the body unchanged, and therefore, the use of alcoholic beverages must involve fruitless wear and tear of the excretory organs, as well as a total waste of the excreted alcohol.

6. That alcohol, if a food, stands apart from all other foods in its want of innocuousness in its relations to the tissues.

7. That a large proportion of the degenerative changes which lead to disease and early death, grow out of the use of alcoholic beverages.

8. That the presence of alcohol in the blood retards the oxydation of effete matters, and gives a putrescent tendency to the system.

9. That, in large doses, alcohol is a substance which narcotizes all animal tissues, and invariably destroys life if administered in sufficient doses, and that the so-called stimulating effects of alcohol in small doses are really finer shades of narcotization, disguised by efforts on the part of the system to excrete an injurious substance and to compensate for its disturbing influence.

With many of the conclusions come to by Dr. Edmunds we heartily concur; and as a general rule, we think that the mass of medical men would be inclined to counsel mankind to lay aside the use of alcohol and other narcotics like tobacco, and confine itself to simple foods and drinks. Men and women drink alcohol because they like the feelings of narcotism; but sooner or later all persons who use much alcohol begin to feel its poisonous effects in impaired energy and deterioration of some of the muscles of the body necessary to the carrying on of life.

Alcohol, of course, should never be used by healthy men, women or children. In disease we come to some other questions; and must leave the power of alcohol in disease to be treated more fully at another time. Suffice it to say, we believe that the alcoholic days of Todd and of his school are at an end, and we trust that, like bleeding and over-purgation, over-alcoholization may never again be in vogue.

Dr. Edmunds belongs to a society of philanthropists who are truly of immense utility in this country and at this moment. Owing to the many discoveries in the useful arts made during this century, we have in this land acquired sudden and unexampled powers of acquiring wealth; but it is by no means so clear that the way in which that wealth is now being spent conduces to the happiness of the nation. On all sides we see disease and deterioration of morals, the result of indulgence in alcohol and tobacco; and the physician of our time, if he possess any sympathy with his kind, will find no surer road to improve the happiness of the race, than by lessening the fatal proclivity of the masses toward alcohol. All those who wish to know the truth of this matter should read this little pamphlet, which is scientific, well composed, and what is more, *true.—Med. Press and Circular.*

